# Measuring embodiment: A review of methods for prosthetic devices

**DOI:** 10.3389/fnbot.2022.902162

**Published:** 2022-12-14

**Authors:** Jacob L. Segil, Leah Marie Roldan, Emily L. Graczyk

**Affiliations:** ^1^Department of Mechanical Engineering, University of Colorado, Boulder, CO, United States; ^2^Rocky Mountain Regional VA Medical Center, Aurora, CO, United States; ^3^Department of Biomedical Engineering, Case Western Reserve University, Cleveland, OH, United States; ^4^Louis Stokes Cleveland VA Medical Center, Cleveland, OH, United States

**Keywords:** embodiment, prosthetic device, prosthesis, outcome measures, ownership, agency, body representation

## Abstract

The development of neural interfaces to provide improved control and somatosensory feedback from prosthetic limbs has initiated a new ability to probe the various dimensions of embodiment. Scientists in the field of neuroprosthetics require dependable measures of ownership, body representation, and agency to quantify the sense of embodiment felt by patients for their prosthetic limbs. These measures are critical to perform generalizable experiments and compare the utility of the new technologies being developed. Here, we review outcome measures used in the literature to evaluate the senses of ownership, body-representation, and agency. We categorize these existing measures based on the fundamental psychometric property measured and whether it is a behavioral or physiological measure. We present arguments for the efficacy and pitfalls of each measure to guide better experimental designs and future outcome measure development. The purpose of this review is to aid prosthesis researchers and technology developers in understanding the concept of embodiment and selecting metrics to assess embodiment in their research. Advances in the ability to measure the embodiment of prosthetic devices have far-reaching implications in the improvement of prosthetic limbs as well as promoting a broader understanding of ourselves as embodied agents.

## Introduction

The experience of having a body is a ubiquitous phenomenon underlying all human experiences. This sense of one's own body, which includes the feeling of being distinct from other objects/persons and the sense of what and where one's boundaries are, is also known as embodiment (Carruthers, 2008; Longo et al., 2008). For most people, this sense of embodiment is automatic and integrates seamlessly with all sensory experiences, thoughts, and behaviors. However, the sense of embodiment can be disrupted by psychological conditions, injuries to the nervous system, or serious injuries to the body, such as major limb amputation. In the case of amputation, embodiment is further perturbed or modified by the addition of a prosthetic limb. Embodiment is of particular interest to the field of prosthetic design since the psychological and physiological integration of the prosthesis within the body has the potential to shape the user's utilization and acceptance of the device (Murray, 2004; Graczyk et al., 2019; Bekrater-Bodmann, 2021).

Embodiment of a prosthesis can occur to varying degrees, but to be fully embodied, all the properties of a prosthesis would need to be processed in the same way as the intact body (De Vignemont, 2011). Embodiment of a limb or device is dependent upon continuous feedback from the tactile, proprioceptive, visual, interoceptive, and vestibular systems (Giummarra et al., 2008), as well as the ability to intuitively control the limb or device (Moore and Obhi, 2012; Caspar et al., 2015; Braun et al., 2018). Prior studies have presented data demonstrating embodiment of existing prosthetic technologies (Schiefer et al., 2016; Graczyk et al., 2018; Petrini et al., 2019), examined the impact of prosthesis embodiment on prosthesis function and use (Maimon-Mor et al., 2020; Fritsch et al., 2021), reviewed the conditions necessary to induce prosthesis embodiment (Ehrsson et al., 2008; Page et al., 2018; Bekrater-Bodmann, 2021), and reviewed continuing challenges for prosthesis embodiment (Niedernhuber et al., 2018; Rognini et al., 2019; Bekrater-Bodmann, 2021).

As surgical techniques and neuromuscular technologies to enable closed-loop bidirectional limb prostheses evolve, the user's experience of the prosthesis and how they use it in relation to the rest of their body will also fundamentally change. For example, recent advances in neural interfaces and neurostimulation have resulted in the ability to provide anatomically appropriate somatosensory feedback from a prosthesis to a user (Raspopovic et al., 2014, 2021; Tan et al., 2014; Page et al., 2018). Additionally, prosthetic control schemes based on neurophysiological recordings of intended movements have also led to improvements in the intuitiveness and reliability of prosthesis control (Raspopovic et al., 2014; George et al., 2019; Lukyanenko et al., 2021; Segil et al., 2021). Measuring how these and other technological advances change embodiment of prostheses is imperative for technology developers, who can use this information to drive design decisions to allow users to regain more fully what was lost.

To this end, prosthesis embodiment must be accurately assessed with reliable and validated measures. However, most existing measures have not been sufficiently tested for their psychometric properties. While many measures for embodiment have been implemented in studies involving preclinical models or able-bodied humans, it is often unclear how they can or should be applied to prosthesis users, who have anatomical, functional, and sensory constraints. This topic is timely, as many research, government, and industry groups have recently increased their interest in the embodiment of prosthesis technology (Bekrater-Bodmann, 2020; Zbinden et al., 2022). In addition, the lack of psychometrically valid measures for upper limb prostheses in particular is a known limitation in the field, and efforts are underway to validate measures of upper limb prosthesis function, psychosocial experience, and disability (Resnik and Borgia, 2012, 2015; Resnik et al., 2013, 2021).

The study of embodiment is multi-disciplinary, and approaches to measuring embodiment span fields and frameworks of inquiry. In this review, we present existing and emerging outcome measures for limb embodiment across the cognitive neuroscience, psychology, neural engineering, and prosthetic design fields of research, and detail how these measures can be applied to measure embodiment in prosthesis users. Frameworks of inquiry in the fields of phenomenology and philosophy were excluded. Rather than detailing requirements for prosthesis embodiment or summarizing prior results of the degree to which existing prosthetic technologies are (or are not) embodied, we focus this review on embodiment measurement techniques and present actionable recommendations for the implementation of these measures in prosthetics research. Our goal is to provide researchers and prosthesis developers practical information about how to measure prosthesis embodiment, so that they can more optimally evaluate rehabilitation therapies, clinical techniques, or prosthesis technology in future studies.

To do this, we first define a model for embodiment which breaks the concept into constitutive domains: ownership, body representation, and agency. We define each of these constructs and explain how they manifest in perceptual experiences and what is known about the neurophysiological mechanisms underlying these experiences. We then describe measures used previously to assess each of these domains, summarize the experimental paradigms associated with each measure, and discuss the benefits and pitfalls of each measure. Finally, we discuss areas for improvement in the measurement of prosthesis embodiment and describe how these measures can be implemented in current research programs, which may promote a better understanding of embodiment in current and emerging prosthetic limb systems. Prior work has taken the crucial step of reviewing embodiment metrics that have been previously applied in prosthetics research (Zbinden et al., 2022). We expand upon this by discussing emerging metrics that can be used, with minor modifications, to further explore embodiment domains, particularly body representation, which has been less frequently studied in the context of prostheses. Expanding the embodiment metrics that can be used in prosthesis research could help researchers and developers more accurately identify limitations in current systems, in order to refine technologies and devices to enhance patient outcomes.

## Embodiment model and definitions

Understanding embodiment can be a daunting task, in part because it is a multifaceted and complex construct, and in part because terminology related to embodiment is often used inconsistently across fields (Longo et al., 2008; Zbinden et al., 2022). We first present a novel model of embodiment and explain how our model relates to prior models presented in the literature to ensure that the reviewed outcome measures can be understood within this framework. We focus specifically on embodiment of the upper limbs, rather than embodiment of the whole body or other parts of the body.

### Conscious vs. subconscious embodiment

**Figure 1:**
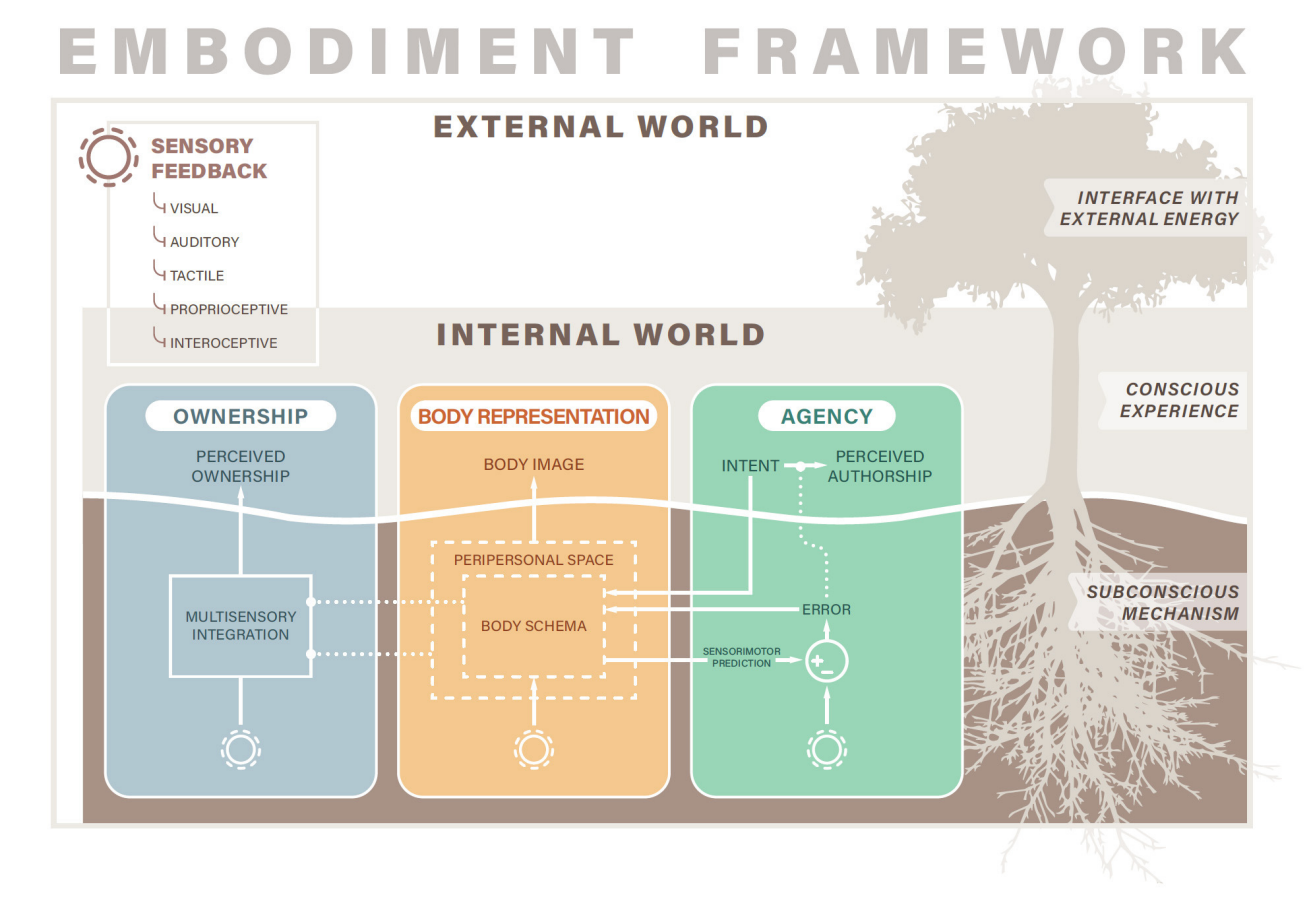
Embodiment framework. The three domains of ownership, body representation, and agency compose the construct of embodiment. Each domain spans both conscious experiences and subconscious mechanisms. Sensory feedback from the external world and internal to the body enter subconscious neurobiological processes that shape conscious experiences of embodiment. Solid lines indicate causal relationships while dotted lines indicate modulatory relationships. While the conscious domains of ownership, body representation, and agency are separable, the subconscious mechanisms interact. For example, the body schema (part of body representation) forms sensorimotor predictions that enter a comparator (circle with + and − signs) that outputs an error signal that ultimately modulates experiences of agency (dotted line). In addition, both the body schema and peripersonal space influence the multisensory integration process (dotted lines) to constrain the types of objects that can be perceived as owned.

Embodiment of a tool or device can refer to both conscious experiences of the device relative to the body and to subconscious neurobiological mechanisms subserving these experiences. In this review, we take the position that the conscious experience of embodiment, or phenomenological embodiment, is rooted in subconscious neurophysiological processes (Arzy et al., 2006; Blanke, 2012; Makin et al., 2017; Braun et al., 2018). In other words, phenomenological embodiment occurs if the tool, device, or limb is represented and processed in neurobiological systems in the same way as intact limbs (Gouzien et al., 2017). As an analogy (Figure 1, right), embodiment is a tree whose roots stretch deep underground and whose above-ground trunk and branches are observable to the passerby. The conscious experiences of embodiment emerge (the trunk and branches) only if supported by the critical subconscious processes operating “below the surface” (the roots). In the sections that follow, we divide each embodiment domain into its conscious and subconscious factors. To explain the conscious factors, we describe the phenomenological experience of each embodiment domain with supporting examples of how it can be perturbed with illusions or how it manifests in disorders. We also provide an overview of the neurophysiological mechanisms operating outside of perceptual awareness (i.e., subconscious or preconscious processes) to support or produce experiences of the embodiment domain.

### The three domains of embodiment

Our model of embodiment consists of three major components: ownership, body representation, and agency (Figure 1). While prior reviews have conceptualized embodiment as consisting of two factors (Zbinden et al., 2022), namely ownership and agency, others have espoused similar views that embodiment consists of three domains (Longo et al., 2008; Bekrater-Bodmann, 2021). We believe that body representation must be included as a separate subcomponent of embodiment, along with agency and ownership, to appropriately contextualize measures relating to body image, body schema, body structural descriptions, and other components of body representation (Longo, 2015). See Discussion section for more details on the justification of our tripartite embodiment model.

Understanding the multidimensional composition of embodiment is important since embodiment measures typically assess one or more of its subcomponents rather than measuring embodiment holistically. Each of the domains of embodiment can be further divided along a conscious/subconscious axis into conscious experiences, actions, and perceptions vs. subconscious neurophysiological mechanisms (Longo et al., 2008).

The three components of embodiment are phenomenologically separable, in that the subjective experience of each domain can occur independently from the other domains. In other words, the experience of each domain can be elicited, modified, or disrupted in isolation without necessarily involving the other domains (De Vignemont, 2007; Longo et al., 2008; Carruthers, 2009; Braun et al., 2018). We have presented examples of specific embodiment illusions and disorders of bodily self-consciousness in the sections that follow to help illustrate the definitions of the three embodiment domains and their phenomenological separability. Prior studies of prosthetic limb embodiment have demonstrated that prosthesis users can experience prosthesis embodiment in individual embodiment domains independently of the other two. For example, depending on the experimental conditions, the prosthesis user can experience agency of prosthesis movements without necessarily experiencing ownership of the prosthetic limb (Marasco et al., 2018).

However, while the three domains of embodiment can be experienced independently, leading to their phenomenological separability, they are not separable on the level of neurophysiological mechanisms (Synofzik et al., 2008a,b; Tsakiris, 2010; Braun et al., 2018; Zbinden et al., 2022). Many of the neural and cognitive processes underlying embodiment play roles in more than one domain, leading to mechanistic interactions across the three domains. The key neurophysiological underpinnings of each domain are presented below, and several of their interactions are depicted in Figure 1 and described in the Discussion section.

#### Ownership

Ownership, or self-attribution, is the belief that a limb, tool, or device belongs to oneself or is “part of my body” or “part of me” (Gallagher, 2000; Braun et al., 2018; Ehrsson, 2020). The conscious experience of ownership involves perceiving that a limb, tool, or device is part of the self, rather than an external, non-self-object. In this review, when we refer to “ownership,” we are specifically referring to upper limb ownership (also called limb identification) as it relates to prosthetic hands and arms being perceived as belonging to the self. Limb ownership can be described as a sub-component of body ownership (also called self-identification), which is the experience that the entire body belongs to oneself (Braun et al., 2018).

The experience of limb ownership is mediated by the subconscious process of multisensory integration (Tsakiris, 2010, 2017; Braun et al., 2018; Ehrsson, 2020), which is the process through which incoming streams of sensory information from different sensory modalities, such as visual and auditory information, are merged and then interpreted as a unitary experience (Stein and Stanford, 2008; Tsakiris, 2010; Ursino et al., 2014) (Figure 1, blue box). Importantly, the sensory information integrated to promote the sense of ownership can be from any combination of modalities, including vision, touch, proprioception, audition, and interoception (Tsakiris and Critchley, 2016; Ehrsson, 2020). To create feelings of limb ownership, multisensory integration requires both temporal synchrony and spatial congruency of the incoming sensory information. When two sensory signals co-occur, there is a window within which a human observer cannot detect any temporal delay and the inputs are perceived to be temporally synchronous (e.g., ~100 ms for visuotactile synchrony; Harrar and Harris, 2005). However, if the delay between the sensory inputs exceeds this window, then the observer can detect the delay and the inputs are perceived as asynchronous (Keetels and Vroomen, 2012; Diederich and Colonius, 2015). Intersensory synchrony is critical for multisensory integration and for creating the sense of ownership. Interestingly, the degree of asynchrony between sensory modalities necessary to abolish the sense of ownership of an object or limb is correlated to the participant's sensitivity to intersensory delay, supporting the role of multisensory integration in establishing ownership experiences (Costantini and Haggard, 2007; Costantini et al., 2016). The experience of limb ownership may also be mediated by internal body maps and other top-down processes, which structure somatosensory input and modulate the multisensory integration process (Tsakiris and Haggard, 2005; Costantini and Haggard, 2007; Ehrsson, 2020).

The sense of ownership is also influenced by expectations and context. To experience ownership of an object, other features of the object, such as its shape, texture, distance from the body, and posture, must be congruent with the limb that it is intended to represent (Braun et al., 2018; Ehrsson, 2020). For example, the sense of ownership is much stronger when experimentally induced for body-shaped objects, such as rubber or prosthetic hands, than non-body shaped objects, such as blocks of wood or tables (Armel and Ramachandran, 2003; Rosén et al., 2009; Tsakiris et al., 2010; Farmer et al., 2012). As another example, the sense of ownership is more likely to be achieved when the object is placed in an anatomically-plausible orientation relative to the body, and is placed within the peripersonal space, which is the space immediately adjacent to the body (Rizzolatti et al., 1997; Tsakiris and Haggard, 2005; Lloyd, 2007; Ide, 2013; Kalckert and Ehrsson, 2014a).

##### Ownership illusion and disruption examples

Ownership illusions provide the opportunity for able-bodied participants to experience ownership of an extracorporeal object, and for researchers to examine neurobiological and perceptual processes underlying experiences of ownership. The rubber hand illusion (RHI) is perhaps the most popular and well-known ownership illusion (Botvinick and Cohen, 1998; Ehrsson et al., 2004, 2008). In the classic implementation (Botvinick and Cohen, 1998), participants are asked to observe a rubber hand sitting stationary on a table, while an experimenter applies tactile stimuli to the hand, such as touches or brushes, while synchronously applying the same tactile stimuli to the participant's own hand, which is hidden from view. The co-location and co-occurrence of the visual and tactile information enable visual-tactile integration that can induce the illusion of ownership of the rubber hand. Note, however, that there are many variations of the RHI task involving different experimental conditions [for a review, see Riemer et al. (2019)].

Alternatively, ownership disruption can be observed in disorders such as somatoparaphrenia, in which a person believes that one of their limbs does not belong to them (Vallar and Ronchi, 2009; De Vignemont, 2011). They may think the limb belongs to someone else or personify it as a separate entity. This disorder is typically seen in patients with brain damage and presents as disownership of the limb contralateral to the lesion (Vallar and Ronchi, 2009).

#### Body representation

Body representation is the knowledge, beliefs, and experiences we have of the physical structure of our bodies, and the cognitive and neural mechanisms by which we dynamically interact with our physical bodies (Gallagher and Cole, 1995; De Vignemont, 2010; Longo, 2015). Body representation is a multifactorial construct, which others have proposed to consist of between two and six subcomponents (Schwoebel and Coslett, 2005; De Vignemont, 2010; Longo, 2015). While the construction of body representation is not yet unified in the field, most authors agree that there are conscious experiences of the body—its size, shape, location, and properties—and unconscious, or preconscious, processes that govern posture and movement dynamically. Even those who propose more than two types of body representation present a means for these subcomponents to be separated along the conscious/unconscious axis. For example, Longo presented six distinct body representations—body image, body semantics, body schema, superficial schema, body model, and body structural description—that were divided along a conscious/unconscious axis into representations that are accessible to conscious awareness, perception, or introspection, and those that operate largely outside of conscious awareness as subconscious mechanisms of action (Longo, 2015). In this review, we will refer to the conscious components of body representation (e.g., body image and body semantics in Longo's formulation) as the “body image,” and the subconscious components (e.g., body schema, superficial schema, body model, and body structural description in Longo's formulation) as the “body schema” (Gallagher and Cole, 1995; De Vignemont, 2010; Longo, 2015) (Figure 1, yellow box).

At the conscious level, the body image is the subjective experience of the size, shape, and physical structure of the body, knowledge about the structure, location, shape, and function of the body and its parts, and the beliefs, attitudes, and emotions we hold about our bodies, which may be influenced by religious, personal, social, and cultural factors (Gallagher and Cole, 1995; Longo, 2015). Statements describing the experience of the body image include: “I have long legs” and “My arms are currently crossed.”

At the subconscious level, the body is represented by the body schema. The body schema is an action-oriented model in the central nervous system that represents the sensorimotor functions and interactions of the body (Gallagher and Cole, 1995). The body schema includes both the forward and inverse internal models of predictive control, as well as predictions about the sensory consequences of actions (Morasso et al., 2015). The body schema includes an understanding of the size, shape, weight, strength, and speed of the body in order to maneuver within the world and localize incoming sensory stimuli (Longo, 2015). The body schema is constructed and modified by incoming sensory information, and in particular proprioception, touch, and vision play critical roles in its formation and maintenance (Figure 1). The body schema operates in real-time during movement to plan appropriate motor commands and continuously receives sensory inputs to update or refine motor outputs (Cardinali et al., 2009; Jovanov et al., 2015). When a tool or prosthesis achieves a similar sensorimotor representation within the body schema as intact limbs, the device is said to be incorporated into the body schema (De Preester and Tsakiris, 2009).

The boundaries of the body are also represented subconsciously, in terms of what areas in space belong to the body, and which areas are adjacent to the body and thus potentially within the realm of the body's control or within range of acting upon the body (Cléry et al., 2015; De Vignemont and Iannetti, 2015; Di Pino et al., 2020) (Figure 1, outer dashed line in yellow box). This body-adjacent space is called the peripersonal space (PPS). PPS is prioritized for attentional processing and is represented differently in the brain than space further away from the body (Maravita and Iriki, 2004; Reed et al., 2006). Incorporation of a tool into the body schema also influences PPS, in that the PPS is reshaped to accommodate the corresponding changes to body representation (Canzoneri et al., 2013).

##### Body representation illusion and disruption examples

The Pinocchio illusion is an example of a body representation illusion and demonstrates how the body image can be distorted by experimentally manipulating coincident sensory stimuli (Schwoebel and Coslett, 2005; Longo, 2015). In the Pinocchio illusion, a participant is asked to touch their nose while receiving vibration of their biceps tendon (Lackner, 1988). Vibration of this tendon provides artificial proprioceptive information that the elbow joint is extending, even though the limb is in fact stationary. The integration of the veridical tactile information from the fingertip combined with the induced sensation of elbow extension causes the participant to feel as if their nose is elongating (Lackner, 1988).

The experience of phantom limbs by people who have had an amputation is a fascinating body representation distortion relevant to prosthesis users and researchers. In this case the limb is no longer physically present, but the body image still includes the presence of an intact phantom limb, resulting in a discrepancy between the physical body and sensory experience (Longo, 2015). Phantom limbs occur in a large majority of people who have undergone a limb amputation (Melzack, 1990; Ramachandran and Hirstein, 1998; Kooijman et al., 2000). Phantom limbs thus demonstrate the relative stability of the body image and its resilience to incredibly large changes in incoming sensory signals from the physical body.

#### Agency

Agency pertains to the self-attribution of actions and the ability to volitionally control a tool, device, or limb (Haggard, 2017; Braun et al., 2018). The conscious experience of agency, also called perceived authorship, describes the feeling that movements and actions of the tool or limb are chosen by the user, initiated by the user, and under the user's control (Synofzik et al., 2008a; Kalckert and Ehrsson, 2012). Statements to describe this experience include: “I am the one controlling my limb” and “This tool obeys my will.”

The sense of agency is thought to arise from the integration of both sensorimotor and cognitive cues (Moore, 2016; Miyawaki and Morioka, 2020). The subconscious sensorimotor mechanism is termed the comparator model (Wolpert et al., 1995; Haggard, 2017; Braun et al., 2018). In the comparator model (Figure 1, green box), intended actions generate both actual motor commands and efference copies that are passed through an internal predictive model that predicts the sensory consequences of those actions (Johansson and Edin, 1993; Wolpert et al., 1995; Flanagan et al., 2003). The prediction is then compared to actual sensory feedback resulting from the action, such as proprioceptive or visual feedback of limb position. If the prediction matches the sensory input, the action is ascribed to be self-caused and the experience of agency occurs (Frith et al., 2000; Farrer et al., 2008; Synofzik et al., 2008a). If the sensory input does not match, the sense of agency is disrupted. Both temporal and spatial congruence between expected and actual sensory input is important for establishing the sense of agency (Synofzik et al., 2008a; Miyawaki and Morioka, 2020). Agency is also modulated by cognitive cues, such as knowledge about action context, mental states, beliefs, or social cues. According to the apparent mental causation theory, the sense of agency is enhanced when a cognitive cue is presented before an action (priority), the cue is consistent with the observed action (consistency), and the self-causation of the action cannot be ruled out by alternate explanations (exclusivity); (Wegner, 2003; Wegner et al., 2004; Miyawaki and Morioka, 2020). In the framework of optimal cue integration, sensorimotor and cognitive cues are integrated together to determine agency, where the relative weighting of each cue depends on its reliability in a given context (Moore, 2016; Miyawaki and Morioka, 2020).

##### Agency illusion and disruption examples

The “helping hands” illusion is an example of an agency illusion and demonstrates how the sense of agency can be influenced by cognitive cues. In this illusion the participant wears a smock that covers their arms and stands in front of a mirror. Meanwhile, an assistant stands behind them such that their arms and hands are visible on either side of the participant in the mirror reflection, in the position that the participant's own arms would normally be. The assistant then performs several actions such as waving, holding up a hand, or spreading their fingers, while the participant observes without making any voluntary motion. When the participant is told what the subsequent action will be (i.e.,—given a cognitive cue), they tend to self-attribute the assistant's actions and experience agency over the observed movements. Interestingly, when the cognitive cue is removed (i.e.,—the participant is not instructed what the next action will be), they do not experience agency of the observed movements (Wegner et al., 2004).

## Outcome measures

This review focuses on ways to measure prosthesis embodiment. We summarize methods that have previously been employed to measure embodiment of prostheses as well as methods that have been applied to measure embodiment in other contexts that may be translatable to prosthetics research. The outcome measures presented below are organized into the three constituent parts of embodiment: ownership, body representation, and agency. While these components of embodiment can be individually measured in different ways, there is no widely accepted method or metric to directly assess embodiment itself. Note also that while many prior studies made claims that a prosthesis technology became “embodied” or that a particular manipulation induced “prosthesis embodiment,” these findings should be interpreted cautiously, given that these studies typically employ measures of individual constructs rather than measures that quantify embodiment directly.

For each measure, an overview of the procedures or experimental paradigms required to implement the outcome measure is presented and followed by a critique of the measure based upon the literature reviewed. References provided are exemplary uses of each measure and are not an exhaustive list. Finally, Table 1 provides an accessible summary of the measures presented in this review and their categorization under our conceptual framework.

**Table 1:** Existing and emerging behavioral (B.) and physiological (P.) outcome measures for prosthesis embodiment.

**Outcome measure**	**Ownership**		**Body representation**		**Agency**		**References**
	**B**.	**P**.	**B**.	**P**.	**B**.	**P**.	
Temporal order judgement tasks							Shore et al., 2002; Azañón and Soto-Faraco, 2007; Moseley et al., 2008; Marasco et al., 2011
Pain perception measurement							Hänsel et al., 2011; Longo et al., 2012a; Mohan et al., 2012; Hegedüs et al., 2014; Martini et al., 2014; Fang et al., 2019
Cross-modal congruency							Pavani et al., 2000; Zopf et al., 2010; Aspell et al., 2013; Blustein et al., 2018; Marasco et al., 2021
Sensory attenuation							Kilteni and Ehrsson, 2017; Fritsch et al., 2021
Skin temperature							Harden et al., 2008; Moseley et al., 2008; Hohwy and Paton, 2010; Kammers et al., 2011; Marasco et al., 2011; Thakkar et al., 2011; Tsakiris et al., 2011; Paton et al., 2012; Rohde et al., 2013; van Stralen et al., 2013; David et al., 2014; Grynberg and Pollatos, 2015; de Haan et al., 2017
Skin conductance response							Lykken and Venables, 1971; Armel and Ramachandran, 2003; Ehrsson, 2007; Ehrsson et al., 2008; Alimardani et al., 2013; D'Alonzo et al., 2015; Braun et al., 2016; Pinardi et al., 2020
Histamine reactivity							Barnsley et al., 2011
Proprioceptive drift							Botvinick and Cohen, 1998; Ehrsson et al., 2004, 2008; Tsakiris and Haggard, 2005; Ehrsson, 2007; Folegatti et al., 2009; Kammers et al., 2009; Longo et al., 2009; De Vignemont, 2010; Rohde et al., 2011; Kalckert and Ehrsson, 2012; Abdulkarim and Ehrsson, 2016; Page et al., 2018; Riemer et al., 2019; Pinardi et al., 2020; Gallagher et al., 2021
Reachability/reaching tasks							Witt et al., 2005; Coello et al., 2008; Bourgeois et al., 2014; Gouzien et al., 2017; Patané et al., 2017; D'Angelo et al., 2018
Limb length and forearm bisection							McDonnell et al., 1989; Giummarra et al., 2007; Sposito et al., 2010; Schmalzl and Ehrsson, 2011; Bolognini et al., 2012; Longo et al., 2012b; Graczyk et al., 2018; Valle et al., 2018; Cuberovic et al., 2019; Engdahl et al., 2020; Bekrater-Bodmann, 2022
Kinematic tasks							Holmes et al., 2006; Cardinali et al., 2009; Kammers et al., 2009
Visual target detection							Graziano and Gross, 1993; Whiteley et al., 2004, 2008; Kao and Goodale, 2009; Di Pino et al., 2020
Cross-hand effect							Yamamoto and Kitazawa, 2001a,b; Sato et al., 2017; Di Pino et al., 2020
Intentional binding							Haggard et al., 2002; Moore and Obhi, 2012; Caspar et al., 2015; Marasco et al., 2018
Questionnaires							Botvinick and Cohen, 1998; Ehrsson et al., 2004, 2008, 2022; Tsakiris and Haggard, 2005; Longo et al., 2008; Dummer et al., 2009; Kammers et al., 2009; Rohde et al., 2011; Walsh et al., 2011; Kalckert and Ehrsson, 2012, 2014b; Imaizumi et al., 2016; D'Angelo et al., 2018; Page et al., 2018; Petrini et al., 2019; Riemer et al., 2019; Rognini et al., 2019; Bekrater-Bodmann, 2020, 2022; Engdahl et al., 2020; Lush, 2020; Maimon-Mor et al., 2020; Roseboom and Lush, 2020; Fritsch et al., 2021; Preatoni et al., 2021; Resnik et al., 2021; Sturma et al., 2021; Lush and Seth, 2022; Slater and Ehrsson, 2022
Qualitative interviews							Murray, 2004; Luchetti et al., 2015; Widehammar et al., 2018; Cuberovic et al., 2019; Graczyk et al., 2019; Middleton and Ortiz-Catalan, 2020
Communicative gesturing							Maimon-Mor et al., 2020
Cortical imaging							Imamizu et al., 2000; Leube et al., 2003; Ehrsson et al., 2004; David et al., 2008; Yomogida et al., 2010; di Pellegrino and Làdavas, 2015; della Gatta et al., 2016; Karabanov et al., 2017; Isayama et al., 2019; Golaszewski et al., 2021

Colored shades indicate the categorization of each outcome measure and exemplary references which describe the measure. Ownership (blue), Body Representation (yellow), and Agency (green).

### Measure selection

We performed systematic searches in PubMed and Web of Science to identify embodiment measures for inclusion in this review. Searches were performed with the combination of search terms “prosthetic and embodiment” or “rubber hand illusion and prosthetic” in April 2021. The two searches across both databases led to 117 total manuscripts after removal of duplicates and irrelevant results. Additional manuscripts were added to the search results as new literature was published on this topic and when the identified manuscripts cited relevant work not found in the original search. Outcome measures were included in this review only if they measured one of the three embodiment domains in our conceptual model and if they (1) had previously been applied to study prosthesis embodiment, or (2) the authors agree that they could be applied to study prosthesis embodiment in the future. In the latter case, the measure will be presented along with recommendations for how to implement it in prosthesis studies. The measures are categorized by embodiment domain and subdivided within each domain into behavioral or physiological measures. While most measures pertain to a single embodiment domain, later sections describe measures that have been used to assess embodiment experiences across domains.

### Behavioral and physiological outcome measures

We have divided the reviewed measures into two broad categories: behavioral and physiological. For the purposes of this review, we have defined “behavioral measures” as those that require perceptions, decisions, or judgements. These measures typically involve verbal responses or voluntary, goal-directed actions undertaken by the participant. In contrast, metrics that do not require these activities are referred to as “physiological measures.” This categorization correlates with our embodiment model in that behavioral measures typically assess conscious experiences, whereas physiological measures typically assess subconscious mechanisms. While behavioral measures may sometimes rely upon subconscious processes, they are categorized as behavioral when the measurement relies on a task that requires a voluntary action, judgement, or decision by the participant. Physiological measures do not require any voluntary action or report and thus more directly quantify neurobiological processes that are occurring outside of awareness.

Note that prior literature on embodiment outcome measures typically categorizes measures as either explicit or implicit, rather than behavioral or physiological. However, definitions for the implicit/explicit terms vary widely across studies (Haggard, 2017; Maimon-Mor et al., 2020; Zbinden et al., 2022), and many studies do not provide definitions at all. This lack of clarity, as well as the use of explicit/implicit to describe other ideas within the field of cognitive science (Longo, 2015), motivated the use of the behavioral/physiological framework presented here. These terms were used by Ehrsson when describing what type of evidence each outcome measure produces during a RHI experiment with upper limb amputees (Ehrsson et al., 2008). Our definitions for these terms focus on operationalizing the conscious/subconscious division in our embodiment framework, as our categorization depends on whether the metric requires access to or awareness of perceptions, experiences, decisions, or actions at the conscious level. Our categorization of measures as either behavioral or physiological focuses on the degree of objectivity of the measure, since our definition for behavioral measures are those which could involve participant bias, whereas physiological measures are those which fully reduce bias.

### Ownership measures

#### Behavioral measures

##### Temporal order judgement tasks

The temporal order judgement (TOJ) task is a classical psychophysical test of sensory perception. The task is to discern which of two sensory stimuli occurs first, and the delay between the stimuli is varied to determine how stimulus timing affects order perception. The TOJ task can also be applied during ownership illusions to quantify changes in ownership during the experimental condition vs. control (Shore et al., 2002; Azañón and Soto-Faraco, 2007; Moseley et al., 2008; Marasco et al., 2011). When implemented to study ownership, the two stimuli are presented in mirror positions on the two hands or arms of the participant. The delay between the two successive stimuli is varied, and subjects are asked to identify the order of the stimuli by indicating the hand that received the first stimulus (or second stimulus during counter-balanced sessions). The point of subjective simultaneity (PSS), which is the delay that yields 50% accurate performance, is the delay at which the participant was maximally unsure about stimulus order because they perceived the stimuli as simultaneous. The PSS demonstrates the relative weighting given to processing sensory input from the two limbs. When the PSS is close to zero, the two limbs are thought to have equal priority in sensory processing. In contrast, inequalities in sensory processing will skew the PSS in the direction of the de-prioritized limb (i.e.,—sensory input from the de-prioritized limb must occur sooner than sensory input from the prioritized limb in order to be perceived as synchronous). Thus, increases in the absolute value of PSS indicate a relative prioritization of one limb over the other, while decreases of the absolute value of PSS indicate symmetry in sensory processing between limbs. Another outcome metric for the TOJ task is the just noticeable difference (JND), which is a measure of sensitivity to delay between the two stimuli, with lower JNDs indicating higher temporal sensitivity.

When measuring prosthesis embodiment with the TOJ task, the hypothesis is that increased prosthesis ownership will result in lower PSS and JND scores as sensory processing becomes more symmetrical between limbs (i.e.,—the prosthesis is owned to the same extent as the intact limb). The assumption when using this measure to quantify prosthetic ownership is that ownership modulates the speed of subconscious multisensory integration, which then improves inter-stimulus delay perception, yielding lower JND values.

The TOJ task has been implemented in a prior study to measure prosthesis embodiment. In this study, a tactor was applied to a region of skin with targeted sensory reinnervation to elicit the sense of touch on the missing hand. The TOJ task was performed after a RHI illusion, in which participants compared the relative timing of touches applied to their intact contralateral limb and tactor input to reinnervated skin. Participants had the greatest shift in PSS during the synchronous conditions when the illusion caused an increase in feelings of ownership of the prosthetic limb (Marasco et al., 2011). In addition to targeted sensory reinnervation, the TOJ task could also be implemented with experimental paradigms involving neurostimulation-based sensory feedback that elicits the sense of touch on the missing limb, or using sensory substitution applied to the residual limb. The comparison stimulus is then presented in a matched location on the intact contralateral limb. Suggested experimental conditions include using this measure with people with amputation without a prosthetic device, with a prosthetic device, and/or after an ownership illusion.

##### Pain perception measurement

Limb ownership can be quantified by the measurement of changes in pain perception, since the speed and accuracy of pain perception is dependent upon one's ownership of their body (Hegedüs et al., 2014; Martini et al., 2014; Fang et al., 2019). Prior work hypothesized that disownership of a limb will decrease perceived pain severity for painful stimuli applied to the limb (Longo et al., 2012a). Building upon this framework, it may be possible to use the change in pain severity on the residual limb of amputees as a proxy of the ownership or disownership of a prosthetic limb. In this measure, pain stimuli can be induced using thermal skin conduction or infrared laser probes. Participants report subjective pain intensity using a scale between 0 (no pain) and 10 (worst imaginable pain), and a staircase procedure is used to find a stimulus with a pain intensity of 5 for the subsequent experiment. This experiment involves an ownership illusion, such as the RHI, in which non-painful tactile stimuli are paired with corresponding visual stimuli to induce the illusion. After the illusion is induced, the pain stimulus, which was previously rated a “5,” is applied to the limb, and the subject responds by reporting the pain intensity. Then, pain intensity can be averaged across experimental conditions to determine if the intensity increased, decreased, or stayed the same as a result of the illusion.

Although this measure has not yet been implemented with prosthetic users, a change in pain perception between the residual and intact limbs could be used to indicate changes in ownership of a prosthesis, especially since the sensory processing of the residual limb is intact above the level of the amputation. A potential experimental method is to quantify pain perception over time as a person with amputation uses a sensorized prosthetic limb system in an at-home setting. At various intervals (e.g., after days of use 0, 15, 30, and 45), the subject would be asked to perform a pain perception measurement across the residual and intact limbs. The hypothesis is that the pain perception of the residual limb would change as the ownership of the prosthetic system increases over prolonged use. This measure could be confounded by phantom limb pain if experienced by the person with amputation, and probably contraindicated as an outcome measure for these individuals. Although some studies have used pain perception to detect changes in limb ownership in able-bodied individuals, the efficacy of the measure can be limited if appropriate experimental controls are not in place (Hänsel et al., 2011; Mohan et al., 2012). The pain perception measurement requires robust experimental controls including the accurate spatial and temporal alignment of pain stimuli across the limbs in the illusion, a rapid application of pain stimuli which does not break the induced illusion, and the use of a calibration routine prior to the illusion to determine the patient-specific pain stimulus to use in subsequent trials. These factors should also be considered if this measure is applied in prosthetics studies.

##### Cross-modal congruency effect

The cross-modal congruency effect (CCE) is based upon an established psychophysical test which requires speeded detection of target stimuli presented in various sensory modalities, such as vision and touch (Pavani et al., 2000; Zopf et al., 2010; Aspell et al., 2013; Blustein et al., 2018; Marasco et al., 2021). Target and distractor stimuli are presented in either the same spatial location (congruent) or different spatial locations (incongruent). The participant is asked to select the location of the target stimulus as rapidly as possible. The difference in reaction time between the trials with congruent stimuli trials and the trials with incongruent stimuli is used to calculate the CCE score. Higher CCE scores indicate that relatively more time was required to respond to incongruent rather than congruent stimuli, which indicates that additional processing was required to ignore the distractor stimulus and respond to the target. The premise is that incongruent stimuli will be more distracting if they are presented to an owned limb or device than if the limb or device is not owned. Thus, the CCE score, as an indicator of relative cognitive processing time, is a proxy for the degree of ownership of the limb. In other words, the CCE score is highest when the feeling of ownership of the limb is greatest.

The CCE task is a measure of ownership but does not itself include procedures for inducing ownership of a limb, device, or tool. Thus, this metric must be paired with a preceding ownership illusion or experimental procedure. To implement this measure properly, the subject must be focused on the discrimination task and all other stimuli should be eliminated from the testing environment.

The CCE measure has been used effectively with prosthetic users in prior studies (Marasco et al., 2021). In these studies, small lights were affixed to the prosthetic thumb and index finger at the same locations as those felt due to sensory stimulation with a sensorized prosthetic system. The prosthesis then receives simultaneous visual and tactile feedback across the index and thumb lights and tactors, and the participant indicates which finger received the touch *via* foot pedal. In this experiment, the visual stimuli are either congruent with the touch position or incongruent (i.e., a distractor). While prior studies have implemented the CCE score to assess the impact of neural interfaces on prosthesis ownership, future work could use this measure in conjunction with sensory restoration provided *via* surgical procedures, such as targeted reinnervation, or with touches applied to the residual limb. The CCE metric may also be useful for assessing longitudinal ownership experiences within patients using sensorized prostheses chronically at home.

##### Sensory attenuation of self-touch

Sensory attenuation refers to the decreased perceived intensity of self-generated touch compared to externally-generated touch (Kilteni and Ehrsson, 2017). In everyday life, sensory attenuation is observed in phenomena such as the inability to tickle oneself. Sensory attenuation has evolved as a critical skill for self-identification in order to differentiate between a non-threatening sensation induced by self-touch vs. a potentially threatening sensation resulting from another person (Kilteni and Ehrsson, 2017). To measure sensory attenuation, a motor applies a force to the participant's finger, and the participant reports the perceived force by pressing on a sensor with their contralateral hand. Prior studies have demonstrated that the reported force is significantly lower for self-produced touches than touches presented by a rubber hand. However, after performing the RHI, touches presented by the rubber hand exhibit a reduction in perceived force that approximates that of self-produced touch, demonstrating ownership of the rubber hand (Kilteni and Ehrsson, 2017).

This metric has already been adapted for use in amputees by Fritsch et al. (2021), who evaluated sensory attenuation while asking subjects to touch their foot with either their intact hand, their prosthesis, or with the hand of another person. The self-touch and prosthesis-touch conditions resulted in significant force attenuation when compared to touches applied by another person. As embodiment of the prosthesis increased, so did the similarity between forces felt under self-touch and prosthesis-touch conditions.

Although this is a promising metric for prosthesis ownership, additional work is needed to validate this metric for use with amputees. In the prior implementation in prosthetics research, the force applied to the foot was not standardized across conditions, and thus could have exhibited systematic shifts due to the method of force application and the person applying the “other-touch” condition. In addition, participants were not instructed on how to move in order to touch their foot. Better clarity in participant instructions and improvement in techniques to report perceived force could lead to more consistent and robust findings. Although attenuation of self-touch is used as a metric for ownership, the mechanisms behind it require a predicted efference copy during self-generated movements, as well as spatio-temporal synchrony between motor and sensory signals. Since prosthesis movement and control are required for this task, this measure is likely dependent on a co-occurring sense of agency and thus may exhibit variability due to the extent of agency. The authors also note that adding in tool-touch as a negative control to compare to self-touch and prosthesis-touch would also help to validate this metric.

#### Physiological measures

##### Skin temperature

Prior studies have explored the relationship between skin temperature of a limb and the ownership of that limb. It is hypothesized that skin temperature decreases with the disownership of a limb (Moseley et al., 2008) and may increase with the ownership of an amputated limb (Marasco et al., 2011). The premise is that skin temperature changes are an indicator for the autonomic behavior of the subject's body, and physiological or artificial limbs that are experienced as owned will display autonomic properties similar to intact bodies and dissimilar to external, non-body objects. Because the skin temperature measure does not require any voluntary action or perception on the part of the participant, we categorize it as a physiological measure.

Infrared thermometers or skin-worn temperature sensors are used to monitor the skin temperature of both the target limb and a control limb (e.g., the contralateral limb) over time (Moseley et al., 2008; Hohwy and Paton, 2010; Kammers et al., 2011; Thakkar et al., 2011; Tsakiris et al., 2011; van Stralen et al., 2013). Then, changes in skin temperature of the target limb are compared to changes in the control limb over the same time interval (Moseley et al., 2008; Hohwy and Paton, 2010; Kammers et al., 2011; Thakkar et al., 2011; Tsakiris et al., 2011; van Stralen et al., 2013). Skin temperature of a target intact limb tends to decrease as it is disowned, while skin temperature tends to increase as feelings of ownership of a limb or prosthetic device increase (Marasco et al., 2011). For people with amputation, the skin temperature of the residual limb is disturbed already, since it is significantly cooler than that of the contralateral intact limb (Harden et al., 2008). Nonetheless, Marasco et al. shows an increase in skin temperature as a RHI induces an increase in ownership of a prosthetic limb compared to a baseline temperature measurement on the residual limb (Marasco et al., 2011). We hypothesize that an increase in skin temperature can occur in the residual limb of a prosthetic user as the person increases their ownership of the limb/device, but additional research is required to answer this question. Well-constrained experimental protocols could implement this measure using an illusion like the RHI and/or measuring baseline skin temperatures before and after prosthetic activity which may increase ownership of the device through regular use.

While several studies have supported the seminal work by Moseley et al. demonstrating the effect of ownership on skin temperature (Moseley et al., 2008), other studies have failed to indicate a systematic relationship between ownership and temperature. De Haan et al. tested the skin temperature drop across 167 subjects in a temperature-controlled room as they disowned their intact limbs and did not show a reliable cooling of the disowned hand (de Haan et al., 2017). Additional studies implemented skin temperature measurement during ownership illusion protocols and did not find significant differences between the experimental and control conditions (Thakkar et al., 2011; Paton et al., 2012; Rohde et al., 2013; van Stralen et al., 2013; David et al., 2014; Grynberg and Pollatos, 2015). This conflicting evidence could have resulted from inconsistent experimental conditions across studies. The skin temperature measure is especially susceptible to uncontrolled variables in the experimental set-up, such as inconsistent room temperature, convective heat loss due to air movement around the subject, and changes in clothing or other insulating materials surrounding the subject. Measurement error related to the accuracy of the thermometer used to make the skin temperature measurement could also lead to inconsistent results. For measuring prosthesis embodiment, additional confounds could be introduced related to the type of socket and suspension, the fit of the socket, heating of the limb due to physical exertion during prosthesis task performance, and other concerns (Niedernhuber et al., 2018). Therefore, the use of skin temperature as a physiological measure of ownership should be used with caution until further evidence is available and should only be used with careful control of potentially confounding environmental variables.

##### Skin conductance response

The skin conductance response is a physiological measure of the change in conductance of the skin from perspiration due to autonomic arousal. The release of sweat from pores on the skin causes a change in electrical conductivity of the skin that can be measured over time or in response to various experimental conditions (Lykken and Venables, 1971). To evaluate ownership, a weapon or action that would cause pain is unexpectedly presented as a threat to the potentially embodied rubber (Armel and Ramachandran, 2003; Ehrsson, 2007), robotic (Alimardani et al., 2013; Braun et al., 2016), or prosthetic hand (Ehrsson et al., 2008; D'Alonzo et al., 2015; Pinardi et al., 2020). Examples of these potentially painful stimuli include hitting the artificial hand with a hammer and stabbing the hand with a needle. The threatening action promotes a sympathetic response, including an increase in sweat production (Armel and Ramachandran, 2003). Skin conductance is typically measured by a pair of surface adhesive electrodes that are placed on the target limb (Lykken and Venables, 1971). The underlying assumption is that if one feels ownership over an artificial hand, then the autonomic response after a threat to that hand will be greater than if no feelings of ownership are present. This measure has been applied to prosthetic users by measuring skin conductance on the residual limb (Ehrsson et al., 2008; D'Alonzo et al., 2015; Pinardi et al., 2020).

The use of the skin conductance response to measure ownership has been widely adopted across the fields of cognitive psychology, neuroscience, robotics, and neuroprosthetics (Armel and Ramachandran, 2003; Ehrsson et al., 2008; D'Alonzo et al., 2015; Braun et al., 2016). The measure is simple to implement and provides a proportional signal to measure the extent of ownership dependent on the level of arousal. Two concerns remain about the efficacy of this physiological measure of ownership: (1) there appears to be participant-dependent variability in responsiveness to this measure, as some participants do not appear to exhibit changes in skin conductance due to threatening stimuli (Alimardani et al., 2013), and (2) the repeated use of threatening stimuli over time may diminish the effect of the threat on sympathetic arousal as the innate surprise decreases.

##### Histamine reactivity

At a cellular level, the immune system identifies what is a part of oneself and what is foreign (Medzhitov and Janeway, 2002). Thus, the reactivity of the immune system within a limb can indicate whether the limb is owned in a physiological sense, with a higher immune reaction indicating that a limb is considered foreign by the body. Barnsley et al. (2011) applied histamine to both a target and a control (i.e., contralateral) limb during an RHI protocol and measured the allergic response. In some studies, saline solutions were also applied to the target limb as another control condition. After inducing the RHI, a larger relative size of the wheal (allergic) response on the target limb compared to the control limb indicated the disownership of the target limb due to the ownership of the rubber hand. The assumption was that disownership of the limb causes the body to respond to the limb as if it were foreign and thus to upregulate the immune response in the limb, causing an abnormally high allergic response.

Measures that involve autonomic functions, such as histamine reactivity and skin conductance, are repeatable, objective, and less susceptible to phenomenological control. While Barnsley's work is cited across the embodiment literature (Blanke, 2012; Seth, 2013; Tsakiris, 2017), the results have not been reproduced widely. It would be beneficial to the field for the histamine reactivity measure to be repeated and validated in future studies. In addition, this measure has not been applied to studies of prosthesis embodiment. This measure could be used to evaluate prosthesis ownership by measuring the difference between the allergic response on the residual limb and the allergic response on the contralateral intact limb. Measurements of histamine reactivity could be compared before and after task performance with the prosthesis. We hypothesize that the immune response to the residual limb would initially be higher than the intact limb, but that the immune response would decrease after prosthesis use if the participant experiences ownership of the prosthesis. This hypothesis is based upon the assumption that the immune system will react similarly in the residual limb of an amputee as the intact limb of an able-bodied individual. Future studies should implement this measure in conjunction with prosthesis interventions to assess this hypothesis.

### Body representation measures

#### Behavioral measures

##### Proprioceptive drift

The proprioceptive drift measure assesses the subject's ability to identify where a limb or body is in space without visual feedback (Botvinick and Cohen, 1998). Since the initial development of this measure by Botvinick and Cohen in their seminal work on the RHI, the proprioceptive drift task has been used in various illusion experiments and similar experimental setups involving prosthetic limbs and/or full body illusions (Tsakiris and Haggard, 2005; Ehrsson, 2007; Folegatti et al., 2009; Kammers et al., 2009; Longo et al., 2009; Kalckert and Ehrsson, 2012; Page et al., 2018; Pinardi et al., 2020).

In this measure, the participant uses proprioceptive feedback to identify the location of their actual hand or limb, which is hidden from view. After the induction of an embodiment illusion, the difference between the actual position of the intact, hidden limb and the perceived limb position is quantified as “drift.” The difference in drift between test and control conditions in the experiment is used to quantify the effect of the illusion and the extent of embodiment of the artificial limb or device. Proprioceptive drift has been used to study embodiment in lower limb prosthesis users through a modification of the task. The participant is asked to point to the position of their phantom limb, and the distance between this position and the position of the prosthesis is measured, with less displacement indicating increased embodiment (Ehrsson et al., 2008; Page et al., 2018).

One key issue with the proprioceptive drift measure is that it is typically measured in conjunction with an ownership illusion and thus is typically categorized as an ownership measure. However, while several studies have demonstrated that proprioceptive drift tends to correlate with the extent of an ownership illusion, as measured by other ownership outcome measures like questionnaires (Botvinick and Cohen, 1998; Ehrsson et al., 2004), we propose that the measure is better categorized as a measure of body representation (i.e., where the limb is perceived to be in space). Supporting this idea, a recent study demonstrated a dissociation between proprioceptive drift and ownership (Gallagher et al., 2021). These and other inconsistent prior results could be explained by differences in the underlying neural mechanisms of ownership and body representation. Specifically, while the proprioceptive drift measure relies on visuoproprioceptive integration, which may include expectations driven by the body schema, it does not depend on tactile information (Rohde et al., 2011). In contrast, the classic RHI relies on visuotactile integration. Therefore, a visuotactile RHI task may not necessarily induce proprioceptive drift. Studies assuming correlation between ownership and body representation without taking these mechanistic differences into account should be regarded with skepticism (Folegatti et al., 2009; Rohde et al., 2011; Abdulkarim and Ehrsson, 2016).

In addition, there are a few methodological concerns with implementation of this measure. The position of the artificial hand relative to the participant's body influences outcomes, such that proprioceptive drift is typically larger when the artificial hand is placed horizontally from the intact hand instead of vertically (Riemer et al., 2019). For use in people with limb loss, the proprioceptive drift measure relies on the perception of a phantom and therefore cannot be used with participants without a phantom in its current form. The way in which the participant indicates the phantom position could also impact outcomes. Work comparing verbal vs. motor indications of limb position in able-bodied subjects showed that variations in how the task is performed could cause different results (Kammers et al., 2009), which potentially indicates a distinction between body schema and body image changes during the RHI. The experimenter should therefore be careful when asking subjects to indicate position of the phantom limb, as a verbal response or pointing gesture may indicate a change in body image, whereas a ballistic pointing or reaching movement using the limb may indicate a change in body schema (De Vignemont, 2010). These differences in methods must therefore be taken into consideration when comparing results across studies.

An advantage of the proprioceptive drift measure is that it can be repeated over time, providing an understanding of long-term changes in body representation in response to continual usage of a prosthesis. In contrast, many other embodiment measures can only occur once during an experiment, making it difficult to assess longitudinal changes.

##### Reachability and reaching distance tasks

Body representation includes an understanding of the lengths of one's limbs and the configuration of one's body. These aspects of body representation can be measured through tasks in which the participant is asked to determine whether they can reach target objects or areas in space. These tasks are behavioral measures because they rely upon the subject's conscious understanding of their own body representation and them making a decision based on this.

In the Reachability Judgement Task, virtual or physical objects are shown in front of the subject, and the subject responds with their judgement as to whether the object is reachable or not (Coello et al., 2008; Gouzien et al., 2017). The Boolean response (yes/no) is collected over a variety of positions of the targets, and reachability judgement and percent error across experimental conditions are compared. In a variation on this paradigm, the Reaching Distance Estimation task asks a subject to identify the point in space at which an object becomes reachable by extending their arm, using a tool, or ambulating (Witt et al., 2005; Bourgeois et al., 2014; Patané et al., 2017; D'Angelo et al., 2018). In a common implementation, an object moves toward or away from the subject, and the subject is asked to stop the object movement when they judge it to be reachable.

Prior literature has used these measures to show how illusions and other experimental protocols can change a subject's perception of their body representation, as well as to study prosthesis embodiment (Gouzien et al., 2017; D'Angelo et al., 2018). In subjects with unilateral amputation, the reaching judgement task or reaching estimation task is conducted with both limbs, and scores are compared between the prosthetic limb and able limb. Participants with poorer prosthesis integration had higher error in reachability judgment for their prosthesis side compared to their intact side (Gouzien et al., 2017), whereas participants with higher prosthesis integration had lower error with their prosthesis side compared to their intact side. The presence of a phantom limb is not required in order to implement this measure since the reachability judgement relates specifically to the prosthesis rather than the phantom limb. The reachability and reaching distance tasks provide an elegant method to quantify changes in body representation, specifically alterations to motor-based PPS.

##### Limb length test and forearm bisection task

Similar to the reachability and reaching distance tasks, limb length and forearm bisection tasks probe the conscious experience of body representation by asking subjects to directly report aspects of the geometry of their limb. The limb length test asks the subject to indicate their total limb length by identifying the end point of the limb, typically defined as the tip of the middle finger (McDonnell et al., 1989; Schmalzl and Ehrsson, 2011; Longo et al., 2012b; Valle et al., 2018; Engdahl et al., 2020). When implemented in subjects with upper limb amputation, participants are asked to identify the end point of their phantom or missing limb, either by pointing to it or indicating the position on a diagram (Graczyk et al., 2018; Cuberovic et al., 2019). Then, the average limb length is used to identify changes in body representation across experimental conditions or due to prosthesis usage.

In amputees, the perceived phantom limb tends to be shorter than an anatomic limb, such that the perceived phantom hand is located within or at the end of the residuum (Longo et al., 2012b). This phenomenon, called limb telescoping, is a pervasive experience for people with limb loss (Giummarra et al., 2007; Longo et al., 2012b). Prior studies have used the limb length test to demonstrate that use of a prosthetic device can cause the perceived limb length to increase such that the phantom hand approximates an anatomically appropriate position comparable to that of the contralateral intact limb (Graczyk et al., 2018; Cuberovic et al., 2019).

A related measure is the forearm bisection task, which requires a blindfolded subject to point to the middle of their forearm (i.e., the midpoint between their elbow and tip of their middle finger; Sposito et al., 2010; Bolognini et al., 2012; D'Angelo et al., 2018). This location is recorded using a digital laser meter or a similar tool, and changes to the perceived midpoint can be tracked during embodiment experiments or illusions. Differences in perceived forearm midpoint across experimental conditions can be compared to examine changes in the body representation. While this test has not yet been implemented in prosthesis research, it could be adapted for use in subjects with an upper limb amputation by asking them to point to or indicate the position of the midpoint of their phantom limb or prosthesis while blindfolded.

The limb length task and forearm bisection task offer simple approaches for assessing changes in body representation, and the limb length test has been previously used in both able-bodied and amputee participants. One drawback to this test is that it can assume the presence of a perceived phantom limb. However, studies have shown that participants without phantom limb awareness still exhibit embodiment of their prostheses (Bekrater-Bodmann, 2022), and thus it would be valuable to modify these task to enable body representation assessment in people without phantom limbs. For subjects without a phantom limb, tests have been used that ask participants to point to the end of their residual with both the prosthesis off and on, or to point to the end of their prosthesis while it is occluded. This past work has shown overestimation of residual limb length when the prosthesis is on, and a similar setup could be applied independent of phantom limb presence (McDonnell et al., 1989). The ability to accurately determine the length of the missing limb or prosthesis could indicate whether the prosthesis is incorporated into the body representation. However, further research would be needed to validate these modified methods before widespread use and to understand differences between prosthesis users with or without phantom limbs.

##### Kinematics tasks

Kinematics tasks involving ballistic reaching or grasping have been performed to evaluate the body schema. Prior studies have shown that these kinds of ballistic actions are altered following tool-use, and in one case following only visual exposure to real and rubber hands (Holmes et al., 2006; Cardinali et al., 2009). Interestingly, ballistic movements do not appear to be affected by the RHI (Kammers et al., 2009). Kinematic tasks differ from the traditional pointing task used in Botvinick and Cohen's RHI experiment in that they require the subject to perform the reach or grasp so quickly that there is no time for the subject to make any on-line adjustments to their movement. Thus, although this task is categorized here as a behavioral task, it likely does not depend strongly on conscious perception or decision making beyond the initiation of the action. Kammers et al. concluded from their study that different sensory modalities are weighted differently for perceptual judgements, which are influenced by the body image, vs. ballistic action, which is governed by the body schema (Kammers et al., 2009). Their results supported the distinction between these two body representations and showed that proprioception has a higher weighting than vision in interactions with the body schema, whereas vision has a higher weighting related to the body image.

Kinematic tasks have been used to assess how the body schema is altered by both the RHI and prosthesis use. Implementations of the task can involve reaching for a virtual or real target from various starting positions (Holmes et al., 2006; Cardinali et al., 2009), using the tip of one hand to reach for the tip of the index finger of the other hand in a single movement (Kammers et al., 2009), and grabbing various objects such as sticks or blocks of different sizes (Cardinali et al., 2009; Kammers et al., 2009). Depending on the task, the outcome metrics can include reaching bias, endpoint error, velocity latency, deceleration latency, maximal amplitude of reaching movement, deceleration peak, or movement time. Thus, kinematic tasks provide rich information about the action-oriented body schema and can be used to assess a multitude of hypotheses about changes to the body schema due to prosthesis use or training.

##### Visual target detection

The body-view enhancement effect describes how the processing of visual stimuli is enhanced with proximity to one's own body (Whiteley et al., 2004, 2008; Kao and Goodale, 2009). Specifically, the reaction time to visual stimuli that appear to be on the body is faster than to stimuli that are not on the body. This phenomenon is thought to arise from the firing of bimodal neurons in the brain which respond to both visual and tactile stimulation (Graziano and Gross, 1993). These neurons have receptive fields which are tied to a specific body part and respond preferentially to visual stimuli placed near this body part. The activity of these neurons is thought to be related to the enhancement of body-related visual processing. Measuring the speed of responses to stimuli at various locations near the target body part can thus produce a map of the brain's interpretation of the physical boundaries of the body.

The Visual Target Detection task quantifies the body-view enhancement effect using a reaction time task in which subjects respond as quickly as possible to visual stimuli presented near their body (i.e., in the PPS) or on their body. Typically, a laser light is projected at various points either on the arm of the participant or at various distances relative to the participant's arm. The subject is asked to press a button as quickly as possible after detecting the visual stimulus, and the average response time is shown to be quicker when the visual stimulus is projected on the participant's arm or hand compared to non-arm areas of space. In able-bodied participants, the reaction time to detect visual stimuli projected onto a tool is initially slower than for stimuli projected onto the body, but reaction times to stimuli on the tool can improve with tool training (Kao and Goodale, 2009). This change in reaction time on the visual target detection task after tool use is thought to indicate increased incorporation of the tool into the body schema.

Changes in body schema after tool use suggests that this measure could also be used to assess changes associated with prosthesis use. In fact, Di Pino et al. previously demonstrated a modified version of this test called a visual-tactile integration reaction time task with an upper extremity amputee (Di Pino et al., 2020). In this experiment, the amputee was shown a visual stimulus approaching them along a table, at which they were seated. Intraneural stimulation was used to deliver a tactile stimulus when the visual stimulus was at varying distances from the subject. The participant was asked to press a pedal as soon as they perceived the tactile stimulus. Healthy subjects performing this test exhibited reduced reaction times when the tactile stimulus was delivered with visual stimuli that were closer to the body. In amputee participants, lower reaction times for visual stimuli close to the prosthesis would indicate incorporation of the prosthesis into the body schema, especially if the reaction times were similar between the intact and amputated sides. Visual stimuli approaching the subject was purposely chosen to be interpreted as potentially more dangerous than a static stimulus. Results could therefore be interpreted to indicate the size of the defense-based PPS, which is the PPS defined by external stimuli that act upon the body, surrounding the residual limb. An enlargement of this type of PPS has been shown to accompany prosthesis embodiment (Di Pino et al., 2020).

##### Cross-hand effect in temporal order judgement

The TOJ task can also be modified in such a way that it assesses PPS in amputees. This was done by Di Pino et al., who asked a participant to perform the TOJ task with their hands either crossed or uncrossed (Di Pino et al., 2020). Two stimuli are delivered to a participant, one to each arm, with a randomly assigned stimulus onset asynchrony. The participant is then asked to identify on which hand they felt the first stimulus. A psychometric curve of these responses can then be used to quantify an esteem accuracy metric, which is the stimulus onset asynchrony corresponding to the inverse of the slope of the psychometric curve. During the traditional TOJ, in which the hands are uncrossed, the participant only needs to rely on somatosensory stimuli to make their judgement about stimulus order. However, hand-crossing introduces disparity between visual and tactile information, and performance typically deteriorates (i.e., the esteem accuracy increases) for able-bodied participants, since tactile somatotopic coordinates conflict with visual external coordinates (Yamamoto and Kitazawa, 2001a,b). This deterioration in performance indicates that the limbs/hands are considered as part of the body, since the disparity between the body-centric somatotopic map and the allocentric external map introduced delays. If the limbs/arms were not part of the body, there would be no body-centric map with which the visual stimuli would conflict. Thus, the cross-hand effect demonstrates incorporation of the limbs into the body representations, and has been previously used to examine the embodiment of prosthetic limbs and some tools (Yamamoto and Kitazawa, 2001a; Sato et al., 2017). This task is thought to impact motor-based PPS since it affects the PPS defined by space that is acted upon by the body. This test has therefore been combined with the visual-tactile integration reaction time test to assess both types of PPS changes (Di Pino et al., 2020).

##### Physiological measures

Cortical imaging techniques to measure body representation are detailed in the Physiological measures spanning domains section below.

### Agency measures

#### Behavioral measures

##### Intentional binding

In normal sensorimotor systems, intentional binding describes the phenomenon in which a person perceives that the sensory consequences of their voluntary action occur sooner than the sensory consequences of an action performed by someone or something else. In other words, a perceptual contraction of time occurs between one's own voluntary action and the external sensory consequences of that action (Moore and Obhi, 2012). Therefore, the presence and extent of intentional binding represents the observer's experience of agency over the action. The delay between action and perception is measured to quantify intentional binding, and by extension, agency of the action.

The relative amount of compression of time between an action and a response can be measured for volitional action and compared to that of non-volitional actions (Haggard et al., 2002; Moore and Obhi, 2012; Caspar et al., 2015; Marasco et al., 2018). To implement this measure, subjects are asked to judge the onset times of different visual events while watching a clock face to measure time (Haggard et al., 2002). Volitional experimental conditions ask the subject to initiate the visual event while involuntary experimental conditions use an automated system to cause the visual event. In another version of the judgement task, the subject voluntarily controls the closing of a virtual hand and determines the delay between observed contact with an object and an auditory cue (Marasco et al., 2018). The judgement error is then calculated as the difference between the actual time from action to response and the perceived time from action to response. Judgement error is then compared between the volitional and involuntary experimental conditions, where enhanced experiences of agency are associated with higher judgement errors (i.e., delays between action and response are judged to be smaller than they really were).

This measure has been successfully applied to prosthetics embodiment research using a neuro-machine interface to provide kinesthetic feedback (Marasco et al., 2018). In this example, the virtual prosthetic hand was volitionally closed by the subjects around a virtual ball, which triggered a physical vibration through a kinesthetic tactor. A tone was randomly delayed by 300, 500, or 700 ms after the virtual contact with the ball, and the amputees were then asked to estimate the delay. Higher agency is indicated when the judged delays are smaller than they really were. In this way, the intentional binding task can be implemented in people with upper limb amputation.

The intentional binding phenomenon is a behavioral measure of agency because it is dependent upon voluntary action. One strength of this measure is that the magnitude of judgement error can indicate the extent of perceived agency, rather than simply indicating the presence or absence of agency over an event. It should be noted that the experimental setup requires precise timing methods in order to reduce measurement error, since differences in perceived delays can be on the order of milliseconds (Marasco et al., 2018).

##### Physiological measures

Cortical imaging techniques to measure agency are detailed in the Physiological measures spanning domains section below.

### Behavioral measures spanning domains

Some types of measures have been used to assess experiences of embodiment across the domains of ownership, body representation, and agency.

#### Questionnaires

Questionnaires have seen widespread use in the study of embodiment because of their relatively simple implementation and analysis. Using questionnaires to measure ownership experiences stems from the original RHI study by Botvinick and Cohen (1998). Since that seminal study, a wide range of similar questionnaires have been developed to support experimental paradigms which probe ownership illusions in able-bodied individuals (Ehrsson et al., 2004; Tsakiris and Haggard, 2005; Kammers et al., 2009; Rohde et al., 2011; D'Angelo et al., 2018) or people with amputation (Ehrsson et al., 2008; Page et al., 2018; Preatoni et al., 2021). Questionnaires are used before, during, and after an illusory experience or prosthesis use to quantify the change in feelings of ownership. In general, the questionnaires are formulated to present both test statements, which refer to feelings of ownership over an artificial hand, and control statements, which do not pertain to ownership and instead are intended to measure the participant's suggestibility or general agreeableness. Subjects respond to each item using a Likert scale to indicate the extent to which they agree or disagree with the statement.

Experiences of agency are also commonly assessed by questionnaire. To study the relationship between ownership and agency, the RHI protocol was modified to incorporate movement into the illusion to create visual-motor integration tasks (Dummer et al., 2009; Walsh et al., 2011; Kalckert and Ehrsson, 2012, 2014b). The questionnaires were also modified or expanded to include items which quantified the sense of agency over the artificial hand. Responses to these agency items, rated on Likert scales, are then compared to responses to control statements. Prior studies have used agency questionnaires to study agency for long-term prosthesis users without the use of the RHI protocol (Engdahl et al., 2020).

RHI questionnaires can also be tailored to different experimental protocols involving rubber, prosthetic, and robotic hands with modifications to question wording. This enables a standard measurement method across diverse applications. Questionnaire scores have also been correlated with other ownership measures to confirm the presence of an ownership illusion (Ehrsson et al., 2004). However, new work has shown that questionnaire responses can be predicted based upon the experiment's demand characteristics (Lush, 2020; Roseboom and Lush, 2020; Ehrsson et al., 2022; Lush and Seth, 2022; Slater and Ehrsson, 2022), which calls into question the utility of these measures. This ongoing debate within the field highlights how it is vital to consider any top-down effects that may influence questionnaire results, sufficiently blind subjects to experimental conditions, and try to account for all confounds. Another limitation of the RHI questionnaires is that they have not all undergone cognitive testing to ensure appropriate participant interpretation or psychometric testing to assess structural validity and reliability. Finally, there is some evidence that the numerical scales and specific words of the questionnaire prompts can skew subject responses even when identical response labels (e.g., “strongly agree,” “strongly disagree”) are used (Longo et al., 2008; Riemer et al., 2019). Therefore, the questionnaire items may be misinterpreted by participants, and the metric may be inconsistently measuring ownership experiences across individuals and experiments.

Beyond the RHI questionnaire and its variants, other questionnaires have been developed to measure psychosocial experiences of prosthesis users, including aspects of embodiment (Imaizumi et al., 2016; Petrini et al., 2019; Rognini et al., 2019; Engdahl et al., 2020; Resnik et al., 2021; Sturma et al., 2021; Bekrater-Bodmann, 2022). For example, the Embodiment scale of the Patient Experience Measure assesses experiences of ownership and some aspects of body image using a Likert scale (Resnik et al., 2021). Resnik et al. assessed the structural validity and reliability of the Patient Experience Measure across a large sample of people with upper limb loss (*n* = 677). Additionally, questionnaires have been developed to explore all three dimensions of embodiment (ownership, agency, location) specifically in prosthesis users, following work performed by Longo et al. (2008). The Prosthesis Embodiment Scale (PEmbS) in particular was developed and validated by Bekrater-Bodmann to explore all three domains in lower limb amputees, although this questionnaire has also been modified for use in upper limb amputees (Bekrater-Bodmann, 2020; Fritsch et al., 2021). Additional questionnaires explore various combinations of these dimensions, such as the Body Image 20 (BIQ-20) and Trinity Amputation and Prosthesis Experience Scales-Revised (TAPES-R; Bekrater-Bodmann, 2020; Sturma et al., 2021). Furthermore, researchers have modified questionnaires that were designed to assess embodiment illusions, such as the original RHI questionnaire, to make them more specific to studying embodiment of prostheses under specific experimental conditions (Petrini et al., 2019; Rognini et al., 2019; Sturma et al., 2021; Bekrater-Bodmann, 2022). While questionnaires are generally prone to bias and inter-participant differences (Maimon-Mor et al., 2020), they are also imperative to the embodiment research field, since they are typically used to validate new embodiment metrics. Until additional behavioral and physiological metrics are developed and validated for prosthesis users, questionnaires are often the easiest to implement and best metrics for assessment of prosthesis embodiment.

#### Qualitative interviews

Qualitative research methods provide a means to understand a participant's subjective experiences with embodiment more holistically. Researchers with backgrounds in phenomenology, psychology, sociology, nursing, and therapy, among others, have used qualitative methods to examine prosthesis embodiment across a wide range of experimental paradigms (Murray, 2004; Luchetti et al., 2015; Widehammar et al., 2018; Cuberovic et al., 2019; Graczyk et al., 2019; Middleton and Ortiz-Catalan, 2020). Qualitative research involves data collected *via* unstructured or semi-structured interviews in which the interviewer asks the participant open ended questions about their experiences, opinions, and thoughts related to embodiment. Example questions include: “Do you feel your prosthesis is part of your body? Or does it feel more like an external tool?”, “How do you experience using your prosthesis?”, “How do you view or think about your prosthesis (Cuberovic et al., 2019; Graczyk et al., 2019; Middleton and Ortiz-Catalan, 2020)?” The data is then transcribed and analyzed with one of several qualitative analysis methods, including Interpretative Phenomenological Analysis (Murray, 2004; Middleton and Ortiz-Catalan, 2020) and Grounded Theory Methodology (Graczyk et al., 2019). The transcribed data is iteratively coded into categories or themes, and in some methods, relationships among the coded data within and across themes can then be used to generate a model that describes the key focus of the research (Strauss and Corbin, 1994, 1998). While qualitative methods are inherently subjective, consensus coding, in which codes are developed and applied collaboratively by teams of analysts, can be used to validate the coding structure and provide robust interpretations of the data (Richards and Hemphill, 2017). The key themes and coding structure often provide insight into the concepts and experiences that are most important or most salient to participants. The themes are often presented with supporting exemplars or quotations illustrating key concepts.

Unlike surveys, which require participants to select from a limited set of options on a set of predefined questions, qualitative interviews allow participants to respond in their own words, to discuss topics that may not have been selected a priori by the researchers, and to shape the discussion to focus on topics or experiences that are most important to them. Furthermore, qualitative methods provide insight into the conceptual relationships among topics from the point of view of participants actually experiencing embodiment illusions or using prosthetic limbs. Qualitative methods can also be useful for hypothesis generation. However, unlike quantitative methods, qualitative methods are not intended to yield conclusions that can be generalized outside of the studied population or to predict future behavior. Thus, qualitative methods are not well-suited for making comparisons across time, across populations, or across experimental paradigms. Nonetheless, these methods are important for the study of prosthesis embodiment and provide theoretical foundations upon which to build future outcome measures.

#### Communicative gesturing

Hand gestures are commonly and universally used while communicating with other people. It is an inherent skill that individuals with congenital blindness use while talking, even though they have never seen them before or learned to emulate them. Since hand gestures are used in everyday life, prior work has evaluated the relationship between the use of hand gestures with the prosthesis during communication and prothesis embodiment (Maimon-Mor et al., 2020). In a prior study, participants were asked to perform a storytelling task and object description task. For the storytelling task, participants were asked to watch a video and then explain it verbally to a listener. For the object description task, they were asked to look at two similar-looking objects and then describe them verbally to a listener. The tasks were designed to encourage gesturing during the relaying of information. Two outcome measures were used: the total number of hand movements made per minute of talking, and a median magnitude ratio (MMR), which reflected how much each arm was used for gesturing. Results showed that amputees and able-bodied subjects did not differ in the number of overall gestures used. However, amputees relied predominantly on their intact arm and made larger motions with the intact arm than the prosthesis, whereas able-bodied gestures symmetrically used both arms. Amputees with a higher embodiment score did, however, exhibit a larger MMR, indicating that their use of the prosthesis in gesturing was more similar to that of the intact arm.

This metric shows promise for use as a behavioral measure for embodiment that would be easy to implement, would require no participant training, and could be used regardless of prosthesis type or the presence of a phantom limb. In this prior study, the relationship between gesture characteristics and embodiment was evaluated using an embodiment questionnaire covering all three domains of embodiment, making it difficult to link this metric to a specific embodiment domain at this time. Additional research is needed to determine which embodiment domain(s) are required for gesturing tasks, and whether each plays a different role in specific gesturing characteristics, such as frequency, range of motion, and type of gesture. Since this experiment was only performed during a short-term study, additional work is also needed to evaluate if this metric could assess longitudinal changes, which would require additional experimental controls.

### Physiological measures spanning domains

#### Cortical techniques and measurements

Non-invasive neuroimaging methods are used to measure cortical activity associated with ownership, body-representation, and agency. Functional magnetic resonance imaging (fMRI), which measures changes in blood oxygen level-dependent (BOLD) signal as a metric of neural activation, can be used to measure the areas of the brain which represent and process information related to ownership or disownership of a limb or device (Imamizu et al., 2000; Ehrsson et al., 2004), body representation (di Pellegrino and Làdavas, 2015), or agency (Leube et al., 2003; David et al., 2008; Yomogida et al., 2010). While inside the fMRI scanner, the participant performs a task or game which relates to feelings of ownership, body representation, or agency. The magnitude of the BOLD signal in various regions of the brain during the task is then compared to BOLD magnitude at baseline to determine which areas of the brain are activated by the visual, tactile, and proprioceptive information presented during the task. The BOLD signal can also be compared between experimental conditions that do and do not induce embodiment experiences to examine hypotheses about the neural bases of these experiences. To understand the relationship between cortical activation and the experience of embodiment, the BOLD signal can also be correlated with behavioral measures such as questionnaires. For example, Ehrsson et al. hypothesized that the premotor cortex and posterior parietal cortex are implicated in the multi-sensory integration process necessary for ownership (Ehrsson et al., 2004), and compared neural activity across synchronous/asynchronous and congruent/incongruent RHI paradigms *via* fMRI. They found that the strength of the ownership illusion during the synchronous and congruent condition correlated with BOLD activity in bilateral premotor cortex. Yomogida et al. found that the supplementary motor area, cerebellum, and some parts of the posterior parietal cortex and right extrastriate body area are active during experiences of agency (Yomogida et al., 2010).

Transcranial magnetic stimulation (TMS) can also be used to study the link between neural activity in the sensorimotor cortical network and the experience of body ownership. TMS can be applied to the primary motor cortex during an embodiment illusion to create motor evoked potentials on demand (della Gatta et al., 2016; Karabanov et al., 2017; Isayama et al., 2019; Golaszewski et al., 2021). Then, electromyography (EMG) measured on the target limb can quantify the motor excitability of the corticospinal circuits during the illusion. The magnitude of the EMG response is compared between experimental conditions to determine the excitability of the neuromotor system while experiencing different degrees of ownership. Studies have shown that the disownership of a limb significantly decreases the motor excitability of hand corticospinal circuits for the affected limb (della Gatta et al., 2016).

There are several limitations to cortical activity measures. First, the equipment necessary to probe these cortical processes is expensive and requires specialized training, which can limit the accessibility of these techniques to only the most advanced research endeavors. fMRI BOLD measurements are based upon large sample sizes and describe sample population statistical parametric maps, which depict probabilities of activation of areas of the brain. The BOLD measure does not give an absolute measurement of the magnitude of an ownership, body representation, or agency phenomenon for a given subject but can be used to indicate differences between experimental conditions or participant groups. In addition, many of the findings related to the neural correlates of embodiment have not yet been replicated in people with upper limb loss, so it is unclear if differences might exist between prosthesis embodiment and general limb embodiment. Despite these limitations, cortical measurement techniques are important physiological measures to help elucidate the neural bases of ownership, body representation, and agency.

## Discussion

For prosthesis users, the phenomenon of embodying the prosthetic limb includes feelings of ownership over the prosthesis, incorporation of the limb into body representations, and experiences of agency of prosthetic movements. While each of these experiences can occur in isolation, they are also constituent parts of the overall experience of replacing the lost limb with the prosthesis, including all ways that this regained limb enables one to interact with and experience the world. This experience of having a voluntarily controllable, self-attributed body that includes the prosthetic limb is the experience of prosthesis embodiment.

This review summarizes existing measures for prosthesis embodiment and emerging measures that can be applied in future studies of prosthesis embodiment. Although most embodiment measures were initially developed to assess illusions in able-bodied individuals, many are applicable in the study of prosthesis embodiment.

Most existing measures of ownership, body representation, and agency are behavioral measures that rely upon conscious perception or action by the subjects. Behavioral measures require the internal translation of subconscious processes into consciously accessible perceptions or decisions, and this translation could be influenced by participant expectations or biases. Furthermore, the way in which these behavioral measures and their associated experimental paradigms are implemented can influence the measured outcomes (Riemer et al., 2019). Well-controlled experimental paradigms are imperative for the appropriate interpretation of behavioral embodiment measures.

Our analysis revealed that prosthesis ownership has the widest range of available measures and has robust measures that are both behavioral and physiological. In contrast, existing measures for body representation and agency are almost entirely behavioral. Thus, the embodiment research field would benefit from additional reliable physiological measures of body representation and agency. To address the existing gaps in embodiment measurement, researchers and scientists across fields continue to develop new measures, often catered to a new application or to explore the underlying subconscious mechanisms of a particular construct. For example, a recent study reported the development of a prosthesis weight perception task to measure body representation in lower limb amputees using a sensory-enabled prosthetic foot (Preatoni et al., 2021). Ideally, all new measures should be assessed for reliability and validity before they reach widespread use. In addition, new physiological measures should be correlated to existing behavioral measures within their embodiment domain to refine hypotheses on the neural underpinnings of conscious embodiment experiences. New measures of embodiment developed for the prosthetics field could also have benefits for embodiment research in other fields. Prosthetics research is an ideal testbed for piloting and refining embodiment measures since the domains of embodiment can be more easily separated for a prosthetic or virtual limb than for an intact limb.

### Body representation as a component of embodiment

The theoretical conceptualization of embodiment remains controversial, and there is no single unified model or definition of embodiment. For example, variability in the definitions of embodiment within the prosthetics field has recently been reviewed by Zbinden et al. (2022).

In this review, we present embodiment as consisting of three constituent parts: ownership, body representation, and agency. However, others define embodiment as consisting of a single part, which is most commonly ownership, or two components, typically ownership and agency (Schofield et al., 2021; Zbinden et al., 2022). Still others separate embodiment into different categorizations altogether, such as perceptual embodiment and motor embodiment (De Vignemont and Farne, 2010), or define embodiment in terms of subconscious processes only (De Vignemont, 2011).

While our three-component embodiment model is novel, prior studies have presented similar tripartite models, which were informed by psychometric analyses of data collected during temporary, experimentally-induced experiences of embodiment or from prosthesis users describing the embodiment of their device (Longo et al., 2008; Bekrater-Bodmann, 2020, 2022; Fritsch et al., 2021). Longo, for example, performed a principal component analysis (PCA) of survey data to construct a model of embodiment and found that the most important factors in the subjective experience of embodiment are ownership, agency, and location. By Longo's definitions of the subcomponents of body representation (Longo et al., 2008; Longo, 2015), the term “location” is a subset of factors encompassed by the broader term “body representation.” Therefore, our tripartite embodiment model is based on these findings but expanded to include other components of body representation beyond self-location. We posit that the more limited concept of “location” emerged from Longo's PCA study, rather than the broader concept of “body representation,” because the analysis was constructed solely from the subjective experiences of the classic rubber hand illusion, which is a simplistic experimental paradigm that does not include many critical aspects of bodily experience, such task-related movement or self-initiated object interactions.

The omission of body representation from other recent models of prosthesis embodiment (Zbinden et al., 2022) may be due, at least in part, to the fact that body representation is a particularly challenging construct to define. Several recent studies have examined the subcomponents of body representation and have not reached consensus about the number of subcomponents or their identities (Schwoebel and Coslett, 2005; De Vignemont, 2011; Longo, 2015). Therefore, prior models may have chosen to exclude body representation to focus on more well-understood concepts. In addition, some authors appear to view efforts to explore the subcomponents of body representation as the formulation of a separate “body representation framework” of embodiment, which is often presented as an alternative to ownership/agency-based frameworks (Zbinden et al., 2022). We do not take this view that body representation must belong to a separate formulation and believe that our tripartite model is one way to harmonize these currently non-intersecting frameworks of embodiment.

Another difference between our embodiment model and prior models is our treatment of the relationship between embodiment domains and subconscious neurophysiological processes underlying these experiences. In our model, we propose that conscious experiences of embodiment (i.e., feelings of embodiment or judgements of embodiment) emerge when the embodied device or object is represented and processed by neural and cognitive systems similarly to native limbs (De Vignemont, 2011). We believe that this relationship also holds within individual domains, such that the experience of ownership of a device emerges when multisensory integration of that device occurs similarly to native limbs, for example. Thus, our embodiment model can be broken into six sub-categories that are the combinations of each of the three embodiment domains (i.e., ownership, agency, and body representation) and each of the two awareness levels (i.e., conscious, subjective experiences vs. neurophysiological mechanisms) (Figure 1). This framework appears to differ from prior embodiment models in which ownership and agency are viewed as being purely conscious, while body representation is viewed as operating only at the subconscious, neurophysiological level (Zbinden et al., 2022). We do not agree with this formulation, since many studies on the subcomponents of body representation demonstrate that some aspects of body representation, such as body semantics and body image, can be felt and experienced through conscious introspection (Longo, 2015).

That body representations contribute to embodiment at the subconscious and preconscious level appears to be less controversial in the field. Indeed, the involvement of the body schema, which is the neurocognitive internal model of bodily actions and their sensory consequences, has long been implicated in embodiment of tools (Maravita and Iriki, 2004; Jovanov et al., 2015). Further, many prior studies discuss how prosthesis embodiment involves incorporation of the prosthesis into the body schema (Giummarra et al., 2007; Preatoni et al., 2021; Zbinden et al., 2022). Therefore, since body schema appears to be implicated in embodiment experiences, it is reasonable that other body representations (e.g., body structural descriptions, superficial schema), and thus body representation as an overall domain, would also be involved in embodiment (Longo, 2015).

As the examples of embodiment illusions and disorders that lead to embodiment disruption presented in the “Embodiment model and definitions” section show, the three domains in our tripartite embodiment model can be experienced independently at the conscious, subjective level. In other words, we posit that these three domains are phenomenologically separable. However, body representation, agency, and ownership are not independent at the subconscious, neurophysiological level. The domains overlap in their sensory inputs and the neural and cognitive processes that support their occurrence. For example, components of body representation play a role in forming ownership and agency experiences. In creating the experience of ownership, knowledge about the body schema and PPS constrains the shape, size, and other spatial properties of the limb or tool that can be ascribed to be part of the self (Rizzolatti et al., 1997; Tsakiris and Haggard, 2005; Lloyd, 2007; Ide, 2013; Kalckert and Ehrsson, 2014a). In promoting the experience of agency, the body schema includes the internal forward models that predict sensory consequences of intended actions (Morasso et al., 2015), which are then compared to actual sensory inputs to determine if the action was indeed self-caused. We propose that the body representation is a modulator of both ownership and agency, and as such, acts as an interface layer that mediates between ownership and agency to shape all embodiment experiences.

We believe that our tripartite embodiment model resolves inconsistencies with other embodiment formulations and has benefits in the organization of outcome measures. The inclusion of body representation as a separate domain of embodiment enabled us to categorize certain outcome measures differently than in prior reviews that presented embodiment as consisting of only two domains (ownership and agency). For example, we categorized proprioceptive drift as a behavioral measure of body representation, while most other reviews categorize it as an implicit ownership measure (Braun et al., 2018; Zbinden et al., 2022). Proprioceptive drift has traditionally been used as an outcome measure for the RHI, which is an ownership illusion (Tsakiris and Haggard, 2005; Costantini and Haggard, 2007; Makin et al., 2008). However, recent studies have demonstrated dissociation between subjective experiences of ownership induced by the RHI and proprioceptive drift outcomes (Dempsey-Jones and Kritikos, 2014; Gallagher et al., 2021). We believe that our categorization of proprioceptive drift as a measure of body representation provides an explanation for these inconsistent results. If proprioceptive drift is actually a measure of body representation, then it is possible that for some experimental paradigms, the illusion both induces ownership experiences and modifies the body representation (thus resulting in high proprioceptive drift), while other implementations only induce ownership without modifying the body representation (thus resulting in low proprioceptive drift). As another example, our tripartite model allowed us to categorize reachability and reaching distance measures as body representation measures (Witt et al., 2005; Bourgeois et al., 2014; Gouzien et al., 2017; D'Angelo et al., 2018). If body representation were eliminated as a domain in our model, it is unclear how these measures should be categorized, as they are not clearly associated with ownership or agency. Thus, we believe that our model of embodiment will have benefits in understanding prior results and in formulating future studies that more accurately assess prosthesis embodiment.

### The role of phantom sensation in embodying prostheses

An aspect that is unique to embodiment experiences in amputees is the interaction of prosthesis embodiment with the phantom limb experience. Phantom limb is very common in amputees, affecting about 85% of those with limb loss (Nikolajsen and Christensen, 2015), and has been found to affect embodiment (Bekrater-Bodmann, 2022). As was noted in some of the metrics above, such as proprioceptive drift, reachability and reaching distance tasks, and the limb length and forearm bisection tests, a phantom limb may be either necessary to conduct the test or may significantly affect the results of these tests. However, other metrics, such as the PEmbS questionnaire, was explicitly designed to not rely on the presence of a phantom and can therefore be used without a phantom limb being present. Additional work is required to assess how the presence or absence of a phantom, as well as the representation of the phantom, may affect the outcome of other metrics. The metrics chosen by the experimenter should therefore be chosen carefully based on the subject population and whether phantom limb awareness (PLA) is present. These inter-individual differences in PLA should also be taken into consideration when comparing embodiment experiences across a population of limb loss participants.

One common assumption made across the literature is that embodiment requires the phantom limb to be co-located with the prosthetic hand, and this co-location, also known as “phantom prosthesis tolerance,” is used as a metric for embodiment. This relationship between embodiment and phantom/prosthesis co-location was investigated more in depth in prior literature through work done with upper and lower limb amputees (Rognini et al., 2019; Bekrater-Bodmann, 2022). Bekrater-Bodmann found that PLA alone was not sufficient for embodiment, and that participants without PLA experienced higher embodiment of their prostheses than participants with PLA (Bekrater-Bodmann, 2022). However, “phantom prosthesis tolerance” helped subjects to overcome this obstacle and was a crucial step in achieving embodiment. This points to the importance of understanding not only the presence, but also the physical representation, position, and behavior of the phantom when evaluating embodiment. This was further supported by another study where all subjects that reported their phantom to be in a position distinctly different from their prosthesis did not report embodiment (Di Pino et al., 2020).

### Selecting embodiment measures in future prosthetics studies

Our intent is that this review will help researchers and scientists select appropriate outcome measures for future studies of embodiment of new prostheses and sensorimotor restoration technologies. To measure the embodiment of a prosthetic device or technology, the researcher should first determine what conscious experiences and/or subconscious processes might be affected by the technology, using the theoretical framework summarized here. This will then enable them to identify which domain(s) of embodiment to assess. For example, if testing a new technology to provide tactile feedback to users of hand prostheses, the researcher might hypothesize that improved tactile feedback could enhance visuotactile integration when observing prosthesis behavior, which might then contribute to enhanced prosthesis ownership.

The researcher must then select an appropriate outcome measure or set of outcome measures to assess the construct of interest (i.e., ownership in this example). The decision to select behavioral or physiological measures of ownership in this scenario could be determined by time constraints related to the experimental setup, availability of specific equipment, or the desire to maximize objectivity. If the researcher wished to measure additional domains of embodiment along with ownership, they could do so, but should acknowledge that there may be no effects in other domains that are not related to the experimental manipulation. Given that body representation may mediate experiences of both ownership and agency, we suggest that body representation measures should be incorporated into future studies of both prosthesis ownership and agency, to allow for future investigations into the mechanistic role of body representation in forming embodiment experiences.

If the researcher wishes to make conclusions about “prosthesis embodiment” broadly rather than about individual embodiment domains, we recommend implementation of sets of measures that include measures of all three domains. For example, the researcher would select one or more measures from each domain and apply them in conjunction during their experimental paradigm. Findings across measures would then need to be integrated in a systematic fashion to make conclusions about “prosthesis embodiment” holistically rather than about ownership, body representation, or agency specifically.

## Author contributions

JS and EG conceptualized the paper, embodiment model, and wrote the original draft. JS and LR performed the literature review. JS, LR, and EG reviewed and edited the manuscript. All authors contributed to the article and approved the submitted version.
